# Immunoglobulin Transporting Receptors Are Potential Targets for the Immunity Enhancement and Generation of Mammary Gland Bioreactor

**DOI:** 10.3389/fimmu.2016.00214

**Published:** 2016-06-10

**Authors:** Xuemei Jiang, Jianjun Hu, Diraviyam Thirumalai, Xiaoying Zhang

**Affiliations:** ^1^College of Veterinary Medicine, Northwest A&F University, Xianyang, Shaanxi, China; ^2^Key Laboratory of Tarim Animal Husbandry Science and Technology, College of Animal Science and Technology, Tarim University, Alar, Xinjiang, China

**Keywords:** immunoglobulin transporting receptors, immunoglobulins, immunity, bioreactor

## Abstract

The functions of immunoglobulin transporting receptors (Ig transporting receptors) in immune system encompass from passive immunity to adaptive immunity by transporting immunoglobulins (Igs) and prolonging their half-life as well as enhancing immunosurveillance. Prior to the weaning, Ig transportations from mother to offspring confer the immediate passive immunity for neonates. After the weaning, FcRn and polymeric immunoglobulin receptor on infant intestinal epithelial cells retrieve Ig in intestinal lamina propria into the gut lumen for preventing pathogen invasion. This is not only improving the pathological consequences of infection but also helping the neonates for developing their own immune response; besides it would be the guidance for designing novel vaccines. Moreover, the investigations on Ig transporting receptors over-expressed transgenic animals have been carried out to improve Ig concentrations in serum and milk; thus, it would be a sustainable method to produce antibody-enriched milk-derived colostrum replacer for neonates. In order to generate mammary gland bioreactor, a series of methods have been developed for enhanced regulation of Ig transporting receptors expression and Ig transportation.

## Introduction

The continuous supply of maternal IgG1 (ruminants) and IgA (monogastric) confers the passive immunity to neonatal mammals until weaning. Neonatal Fc receptor (FcRn) and the polymeric immunoglobulin receptor (pIgR) are responsible for the transportation of these maternal immunoglobulins (Igs). FcRn has been considered as a saturable receptor that mediates the passive transfer of IgG from mother to offspring and protects IgG from catabolism (1). pIgR is responsible for the transport of pentameric IgM and/or dimeric IgA by binding with their joining peptide chain (J-chain) (2). The binding affinity of pIgR toward IgM and IgA is differing among the species; high affinity could be found in human and bovine compared to mice and rats (thereby less IgM and IgA secretion in the milk) (3, 4). The expressions of FcRn and pIgR in mammary gland vary among different animal species and influence the level of Igs in milk. For instance, IgA constitutes 90% of total milk Igs in monogastric species, but it is only 9% in case of bovine (5).

The Igs transport from mother to offspring occurs through placenta and small intestine; these two sites exhibit different significance in different animals (1, 6, 7). Passive immunity relies on prenatal IgG transfer mediated by FcRn on placental syncytiotrophoblasts in monogastric animals, whereas in ruminants, it relies on Ig absorption from colostrums after birth (1, 8). It has been reported that the FcRn expressed on antigen-presenting cells (APCs) could bind with antigens; then, the immune complexes are delivered to dendrtic cells (DCs) for primary immune response (9, 10). In addition, the pIgR on the mucosal surfaces could translocate the secretory IgA (SIgA) from the lamina propria into intestine and maternal precursors of IgA-containing cells would home to the mammary gland where they secrete IgA into milk (11–13). Thus, the FcRn and pIgR could protect animals from the pathogen invasion in intestinal, respiratory and reproductive systems, and/or dietary antigens (1, 12). Hence, both FcRn and pIgR play important roles for not only strengthening passive immunity but also promoting active immunity. It has also been observed that significant increase in humoral responses and mAbs production without any sign of autoimmunity in the transgenic (tg) mice (14–16).

**Figure 1:**
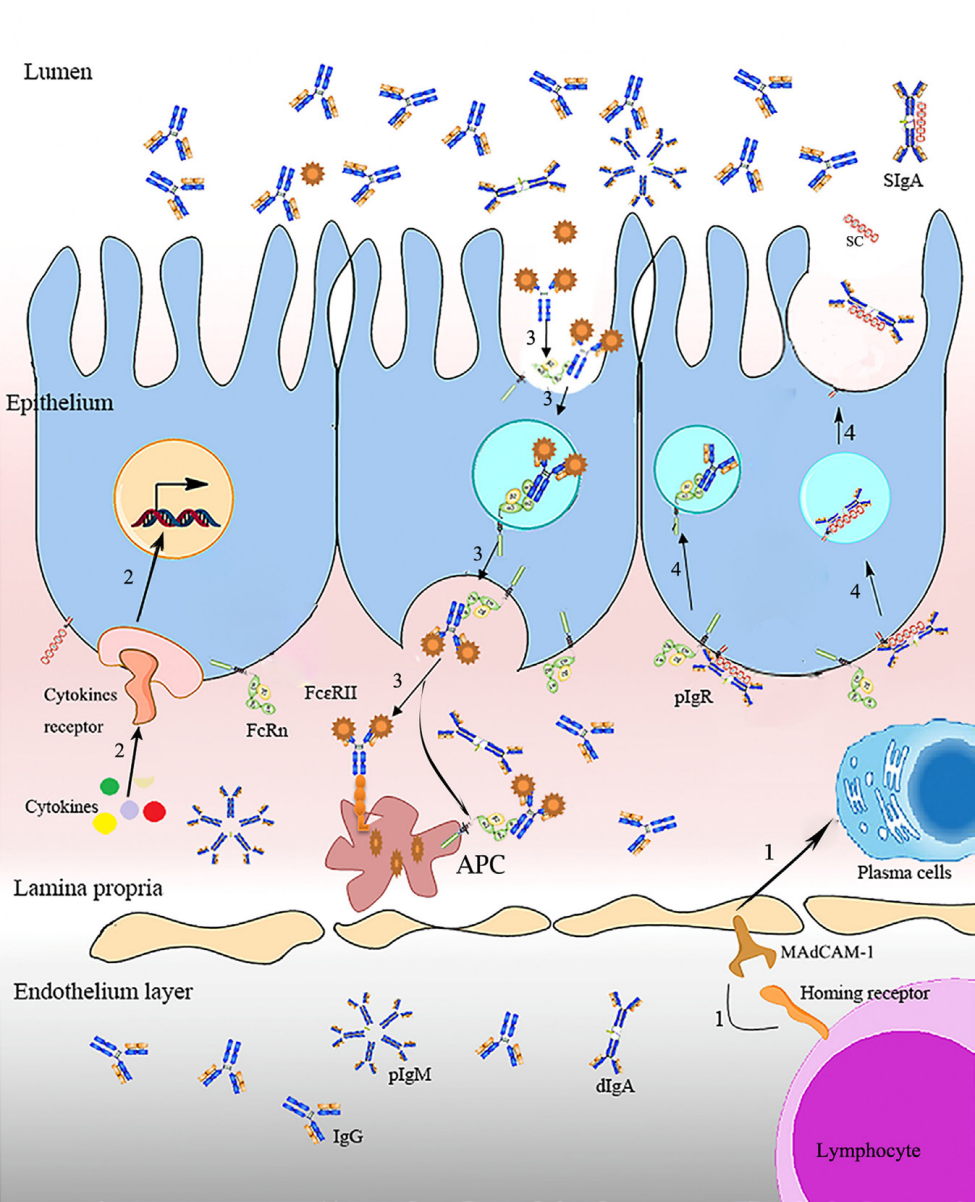
**Regulation and surveillance of FcR on immunity**. IgA antibody-secreting cells homing receptor (LHR) on lymphocyte homing to diverse mucosal surfaces, binding with vascular addressinMAdCAM-1 (mucosal address in cell adhesion molecule-1), VCAM-1 (vascular cell adhesion molecule-1), and the mucosal epithelial chemokine CCL28, mediates lymphocytes homing when there is pathogen invasion, a process that enables the passive transfer of maternal IgA antibody from the mother to the gut of the immunologically naive newborn (17). The interactions of signaling proteins activate *FcR* transcription. Molecular containing IgG-Fc (e.g., antigen–antibody/Fc complex) is internalized into acidified endosome (endocytosis) by binding with FcRn on the mucosal surfaces, and presented to APC. Ig transporting receptors mediate Ig transcytosis from the basolateral into the lumen (1, 11).

Numerous studies have reported that multi-copies of FcRn and pIgR in animals could exhibit an increased ability in strengthening immunity. Besides, the strategies of genetic engineering and molecular regulations have been applied to regulate the expression of FcRn and pIgR. Factors involved in the regulation of passive immunity transfer and immunosurveillance are summarized in Figure 1. This review aims to summarize the vital roles of FcRn and pIgR for the improvement of passive immunity and adaptive immunity; as well as the strategies coupled with the regulations of FcRn and pIgR.

## Passive Immunity Improvement Mediated by FcRn or pIgR

Ruminants are born hypogammaglobulinemic (reduction in all types of gamma globulins). The young ungulates are depending on mammary secretion of colostrum and intestinal absorption of Igs prior to the development of their own humoral defense system for enhancing intestinal and systemic protection (18). In general, a minimum of 150–200 g of IgG is required for achieving adequate passive transfer within 2 h after birth (19). A failure of passive immunity delivery will occur when a threshold concentration of IgG is not reached before closure occurs (20). Moreover, in a mice study, lacking of maternal antibodies led to delayed generation of their own lgG compared to the normal littermates (21). As such, the intake of antibody-enriched milk (for the neonates) is necessary for the establishment of animals’ adaptive immune defense in early life. Since milk is a convenient source of antibody collection, bovine is the superexcellent choice used for polyclonal and monoclonal antibody production. In FcRn over-expressed tg rabbits immunized with ovalbumin, a long serum persistence of IgG (7.1 ± 0.46 days when 5.3 ± 0.3 days in control group) and the highest IgG level [31.61 ± 2.7 mg/ml while 14.8 ± 2.6 mg/ml in wild type (wt) (*p* < 0.01)] were observed (22). In addition, it has been observed that more IgG were transported into milk after upregulated the expression of FcRn receptors in the mammary glands of possum, rabbit, and bovine (23–25). On the other hand, continuous milking is defined as a management system without a planned dry period; it has been described to reduce health problems common in periparturient cattle, but resulting in reduction on colostrum Ig and subsequently calf health (26–28). In FcRn over-expressed tg bovine, the increased IgG protection and transportation could be achieved with adequate IgG along with continuous milking; thus, it reduces the health problems associated with the high yielding transition cow. FcRn or pIgR over-expression in bovine could also be useful for the treatment of mastitis. In general, the control of mastitis during lactation relies on administration of antibiotics or non-steroidal anti-inflammatory drugs; nonetheless, the antibiotic treatment is inefficient and the antibiotic residues would pass on to milk (29). The tg bovine over-expression of pIgR could increase the SIgA in the mammary gland; it would provide stronger and longer protection against the mastitis pathogens from the environment. The free secretory component (SC), the extracellular ligand-binding region of pIgR, is an important component of innate anti-microbial defense (11). In addition, IgA-containing cells would home to the mammary gland when there is inflammation, increased pIgR in mammary gland of tg bovine could transport more SIgA to resist environmental pathogens as well as continuous supply of Igs into milk. Ig-enriched milk from tg animal is an effective alternative to antibiotics, the widespread use of antibiotics alternatives would reduce antibiotic resistance, accordingly, to better maintain the intestinal homeostasis, especially to the newborn (30, 31).

## Adaptive Immunity Improvement Mediated by FcRn and pIgR

The presence of FcRn on APCs suggests that FcRn may influence the antigen presentation (32, 33). Generally, vaccines exhibit a shorter half-life (several hours) but immunogen containing IgG-Fc exhibits longer half-life (20 days) with the confirmed involvement of FcRn on enterocytes and APCs; further it could reach up to 30 days in FcRn over-expressed animals (34). The immunization of FcRn over-expressed tg mice led to 3- to 10-fold increases of antigen-specific IgM and IgG in serum, as well as higher number of antigen-specific B cells and DCs in spleen (16). The elevated antigen-specific IgM and IgG levels were proposed to be the result of the increased diversification of the antigen-specific Ab repertoire (35). bFcRn tg mice immunized with a conserved hemagglutinin subunit 2-based synthetic peptide mounted a robust immunoreaction on day 28 that continued to rise through day 50 while wt mice showed a weak immune response (14). The strength humoral immune response was owing to a higher level of ICs and their increased phagocytosis by the tg neutrophils (NE) and greater influx of these cells into the regional secondary lymphoid organs (35, 36). In another study, tg mice expression of bovine FcRn (bFcRn) in secondary lymphoid organs can boost a threefold of antigen-specific activated T helper (Th) cells compared with wt immunized with T-dependent antigens (9). Hence, tg DC can phagocytose and present antigens to Th cells more efficiently when loaded with Antigen–IgG ICs (35, 37). Meanwhile, ligation of FcRn to ICs can also induces the production of IL-12 from DCs, thereby activating CD4+ T(II) cells in the induction of Th1 polarization and priming CD8+ T(I) cells in the promotion of cytotoxicity activation (37).

In general, the antibodies must be active at the portals of viral entry in the gastrointestinal tract to prevent intestinal infection. SIgA, produced by selective transport of pIgA across mucosal epithelial barrier by pIgR, is the first line of specific immunological defense against environmental pathogens. During the adaptive immune response, the immune system could prime pIgR for the transportation of IgA produced in the laminar propria into intestinal tract as SIgA (6, 12). It has been proved that pIgR is involved in lymphocyte homing in addition to transporting mucosal pIgA (11). pIgR knockout mice lack mucosal Igs and accumulate 100-fold of serum IgA than wt, meanwhile, 14-fold IgA-secreting plasma cells was detected in the intestinal lamina propria compared to wt (38). Increased lymphocyte and SIgA in mucosa help to maintain mucosal homeostasis in the intestine of the neonate. FcRn also behave an important factor on bacterial colonization and pathological consequences of infection in addition to antigen presentation. Tg mice with human FcRn exhibited that antigen within the lumen can be retrieved by administering specific IgG intravenously. These formed Antigen–IgG ICs are retrieved by the epithelial cell and transported into the lamina propria, being internalized by APCs (39, 40). In a *Helicobacter heilmannii* infection model, the specific IgG were exclusively presented in gastric juice of wt mice, while lymphoid follicles and bacterial loads have increased along with deeper gastric epithelium invasion in FcRn-deficient mice (41). FcRn over-expressed tg mice fed with *Francisella tularensis* led to more efficient antigen recognition in the gastrointestinal tract and mucosal localization enhancement that confers immune protection (42). Similarly, the FcRn-mediated transport of IgG across the gastric, genitourinary, and lung epithelium is associated with protection from viral infections and *H. heilmannii* at these sites (39). Likewise, the FcRn is the vehicle that transports luminal antigens across the luminal epithelial barrier and presents the cargo to related immune cells. The application of mucosal vaccination targeted to FcRn can effectively promote the internalization of immunogen, resulting in substantial and enabling immune efficiencies. The non-immunized 7- to 8-month-old bFcRn tg mice did not show detectable antinuclear antibodies with the same general antibody profile compared to wt littermates (43). Hence, tg animal with bFcRn over-expression could be proposed as an ideal choice for monoclonal antibody production; because it enhances immune responsiveness without eliciting autoimmunity (14). However, the vaccinated wt and pIgR knockout mice behaved equally resistant despite dramatic differences in the titer of SIgA in intestinal secretions in a *Salmonella typhimurium* challenge experiment without any differences in terms of CD8+ T cells and T-cell responses (44). In another *S. typhimurium* challenge experiment, pIgR knockout mice were profoundly sensitive to infection with *S. typhimurium* and shed more bacteria that can readily infect other animals (45). These findings suggest that the major role of pIgR probably to resist the invasion of mucosal antigens based on SIgA, rather than protection of local mucosal surfaces by prompting an immune response.

## Current Understanding about the Regulations of FcRn and pIgR

In order to increase the host immunity and antibody production, a series of methods have been developed for enhanced regulation of FcRn and pIgR expression. It will bring substantial advantages for the production of antibody-enriched milk, which would replace colostrums and serve as functional food.

### Genetic Modulations

Gene polymorphisms and haplotypes of receptor genes are the crucial factor to the antibody production and livability of the neonates (41, 46–48). In order to harvest antibody-enriched colostrum or milks, Ig bioreactors could be bred by genetic engineering with respect to FcRn and pIgR on the basis of their polymorphisms and haplotype research. This would also be useful in the intervention to some immunosuppressed periparturient cows. Besides, the development of nuclear transplantation technique and the CRISPR/Cas9 system (clustered regularly interspaced short palindromic repeats/CRISPR-associated system) realize the possibility for using cattle as an attractive candidate of bioreactor.

Allelic variation in *FCGRT* (which encodes the α-chain of FcRn) is associated with variation of IgG concentration in neonatal calves. Among five different variable number tandem repeat (VNTR1–VNTR5) in *FCGRT* promoter, the monocytes from VNTR3 homozygous individuals express 1.66-fold more FcRn transcript and show an increased binding to polyvalent human IgG when compared with monocytes from VNTR2/VNTR3 heterozygous individuals; VNTR3 allele supports the transcription of a reporter gene twice as effectively as the VNTR2 allele; moreover, monocytes from VNTR3 homozygous individuals were reported to bind IgG at acidic pH more efficiently than heterozygous individuals (47). β_2_-microglobulin (β_2_M) exons II and IV are identified with 12 single-nucleotide polymorphisms (SNPs) and were assorted into 8 haplotypes. One of the haplotypes (the β_2_M 2, 2) showed an increased risk of failure for Igs transfer (47). Researchers have also identified three divergent haplotypes of pIgR and explained the variation in the concentration of SIgA and the pIgR level (46). Genome-wide analysis identified a significant association between SIgA and six SNPs located in the PIGR, PIGR-2, PIGR-5, PIGR-9, PIGR-13, PIGR-17, and PIGR-19. Pair-wise analysis demonstrated that all six SNPs were in almost complete linkage disequilibrium. PIGR-17 transformed to alanine from valine at codon 580, and PIGR-2 located in the promoter region is likely to influence the quality or quantity of the gene product (49). IgG clearance is more rapid in β_2_M-deficient mice than in α-chain-deficient mice (23, 50, 51). However, the homologous molecules of β_2_M, such as MHC-I, are not known to extend the half-life of IgG. There must be another β_2_M-related molecule that plays a supporting role during the recycling of IgG. These results suggest that supplemental copies of the gene may prolong half-life of IgG as well as maintain a high IgG concentration. A study using FcRn tg mice in mammary glands results in an increase of IgG levels both in milk and serum (23). Another study reported that, tg mouse over expressing bovine *FCGRT* led to a higher transcription and expression of FcRn and an extended IgG half-life (52). Furthermore, the tg mice over-expression of pIgR from 60- to 270-fold above normal pIgR showed total IgA levels in milk to be 1.5- to 2-fold higher compared with IgA levels of wt mice (53). An overview of the genetic modulations on Ig transporting receptors studies associated with transcytosis is shown in Table 1.

**Table 1:** **Genetic modulation on FcRn or pIgR**.

Receptor	Target gene	Genetic modulation	Species	Experimental outcomes	Reference
FcRn	*FCGRT*/*β2M*	*FCGRT*/*β2M* over-expression	Murine	Twofold serum IgG increase in milk and two- to fourfold increase in serum	(23)
	*β2M*	*β2M* disruption	Murine	10^4^-fold IgG reduction in serum	(21)
	*FCGRT* or *β2M*	*β2M* knockout or *FCGRT* knockout	Murine	Decreased IgG half-lives in β2M-deficient mice (21.8 h) and *FcRn FCGRT*-deficient mice (26.6 h)	(50)
	*β2M*	*β2M* over-expression	Brushtail possum	Increased FcRn transcription and IgG concentration in milk	(24)
	*FCGRT*	*FCGRT* over-expression	Murine	Three- to ten-fold increases of antigen-specific IgM and IgG, which lead to twofold increase of specific titers in the hemagglutination inhibition assay	(16)
pIgR	*PIGR*	*PIGR* over-expression	Murine	1.5- to 2-fold IgA increase in milk	(53)
	*PIGR*	*PIGR* over-expression	Murine	10- to 270-fold (0.1–2.7 mg/ml) SC protein increase in milk	(54)
	*J chain*	*J chain* disruption	Murine	Stable binding of pIgA and SC decreased	(55)
	*PIGR*	*PIGR* knockout	Murine	Lack of active external IgA and IgM transcytosis completely	(54)
	*rab3b*	*rab3b* over-expression	Epithelial cells	Ten percent reduction of dIgA transcytosis	(56)

Genome engineering techniques are targeting Ig transporting receptors for improving the Ig concentrations in milk and maintain immune homeostasis, thereby enhancing the chance to generate Ig bioreactor by combining natural genetic variation selection.

### Molecular Modulations

**Figure 2:**
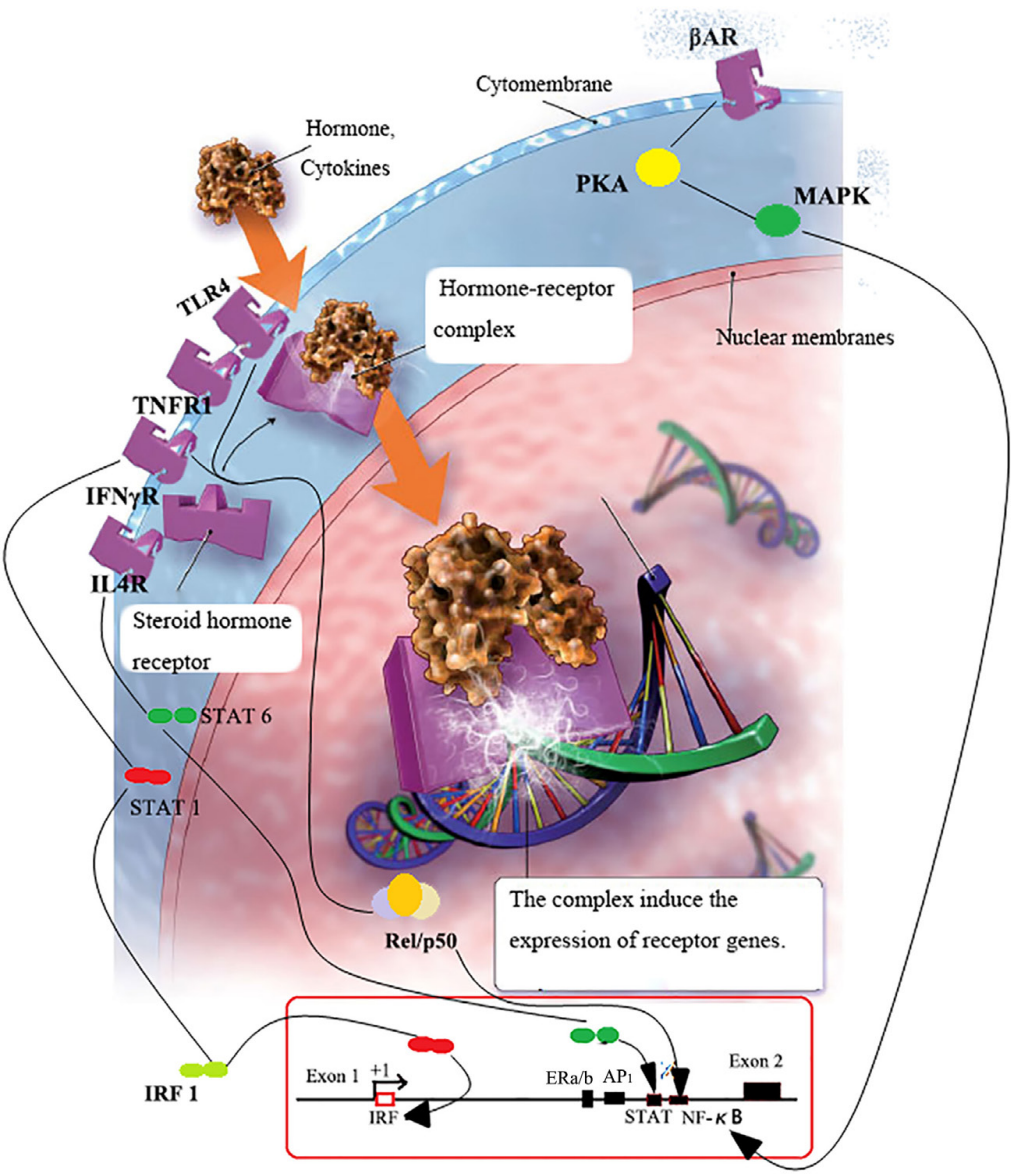
**FcRn or pIgR transcription regulation**. Serum NE binds with β2AR on B cells and activates PKA–p38 MAPK pathways, which subsequently leads to the release of bound p-p38 MAPK to bind with enhancer in the promoter region of *pIgR*, and pIgR increase followed (62). When inflammation occurs, lymphocyte homing induced antibodies production in lamina propria; subsequently, microbes bind with epithelial TLRs with MAMPs and activate NFκB; cytokines released in the vicinity of lymphocytes bind to cytokine receptors and activate IRF-1/NFκB-dependent pathways (58); IRF-1/NFκB proteins get into nucleus and trigger the transcription of Igs receptors (63). As a consequence, concentrations of Ig transporting receptors increase.

Numerous pieces of evidence suggest that microbial-associated molecular patterns (MAMPs) stimulate expression of pIgR on IECs, as part of a homeostatic loop in which the microbiota enhances the production of SIgA, which in turn regulates the composition and function of the microbiota (57, 58). Ultraviolet-inactivated reovirus induced a stronger increase in pIgR expression than live virus in HT-29 cells, suggesting that the induction of pIgR expression required viral components but not viral replication (59). It has been reported that MAMPs stimulate the expression of pIgR, more specifically, the immune response was initiated by microbial component, the toll-like receptor ligands, and then induced *de novo* synthesis of RelB and activation of *PIGR* transcription through the TLR3 pathway (11). The regulation pathways are presented in Figure 2. Additionally, in PRM/Alf mice with a huge extended intestine, a twofold increase of IgA-containing cells and pIgR expression in mammary gland as well as two- to fourfold increase of IgA in milk have been described compared with C57BL/6J mice (29). This result indicated that the intestine can export IgA-containing cells to the mammary gland; and MAMPs could be a stimulation of pIgR expression and SIgA accumulation in mammary gland and milk. As such, MAMPs can be applied on the Ig transport receptor tg cattle to acquire immune milk with enriched antibody. Additionally, the signals (released by antibody and microbiota in neonate gut) have a modulation effect on the maturation of intestinal barrier function and activation of endogenous Ig transportation, as well as the development of adaptive immunity (60, 61).

Host cytokines, such as IFN-γ, IL-4 and TNF-α can regulate the gene transcriptions of Ig transporting receptor by activating and nuclear of STAT1 dimers, resulting in de novo transcription of AP-1, NF-κB, and IRF-1 (58, 64) (Figure 2). For instance, ISRE presents at the first outer exon in promoter region of FcRn (the binding site of IRF-1). It has been reported that the stimulation of bovine endothelial cells with the IFN-γ could lead to the appearance of activated STAT1 in the nucleus and increased transcription of the IRF1 gene. This has resulted in rapid upregulation of the bFcRn expression in endothelial cells (65). Interactions among these cytokine-inducible elements in exon 1 and intron 1 of the PIGR gene may be responsible for the observed synergy between IFN-γ, IL-4, and TNF in upregulation of pIgR expression (58, 66). One putative NFκB transcription binding sites was identified in the 5′-flanking region of the mouse FCGRT promoter and was the close proximity to the transcription start site (63). A fourfold increase of bFcRn gene in spleen, and twofold increase of bFcRn protein in macrophages were observed in the bFcRn tg mice stimulated by intraperitoneal LPS injection (63). The activation of NF-κB by inflammatory cytokines could enhance the gene transcription of FcRn indirectly by synergizing with STAT1 to activate IRF1 gene transcription, or by binding to a cognate element in the 5′-flanking region of the FcRn genes directly (63, 65, 66). Pro-inflammatory agents can induce the rapid and temporary upregulation of bFcRn; it could be the immunologic adjuvants and optimize the expression and function of Ig transporting receptor in the professional APCs, this contributes to the much augmented humoral immune response.

The concentrations of FcRn are significantly upregulated in late pregnancy and reach their peaks during colostrogenesis; then downregulated in the lactation period and coincident with the Ig concentrations in milk and colostrum that differs at various stages of lactation with the highest concentrations appearing in early colostrum secretions (5). In an *in vitro* study, the stimulation of bovine mammary epithelial cells with estrogen (E2) and progesterone (P4) induced an increase of FCGRT mRNA, coincident with FcRn expression during late pregnancy and the colostrogenesis period; meanwhile, a similar increase was observed on bRab25 and bRhoB mRNAs (members of GTPases) and assist recycling or transcytosis (67). This reveals the regulatory mechanism of E2/P4 for colostrogenesis extended temporarily by increasing the expression of FcRn and transcytosis-related motor proteins. However, the binding sites of E2/P4 have not yet identified in FcRn gene.

## Conclusion

The clear understanding of the mechanisms behind the expression and mediation of Ig transporting receptor in the mammary gland and intestinal mucosa would contribute for the better intervention of Ig transfer from mother to fetus. This could also aid for the development of mammary gland bioreactor. Ig transporting receptors could mediate the immunity transfer and immune surveillance, thereby the high survival rate and improved immune system can be achieved. In Ig transporting receptors over-expressed tg animals, the increased Ig concentrations in serum and mucosa can supply adequate Ig through continuous milking as well as safe and effective protection against mastitis pathogens. Moreover, the presence of Ig transporting receptors in mucosal epithelium is capable of transporting antibody or antigen–antibody complex bidirectionally, and further present to APCs. This would help to design immunization strategies for mucosal protection from infections.

## Author Contributions

XZ, XJ, and JH contributed to conception and design; XJ contributed to acquisition of data and manuscript writing. XJ and JH made equal contribution to this paper.

## Conflict of Interest Statement

The authors declare that the research was conducted in the absence of any commercial or financial relationships that could be construed as a potential conflict of interest.

## References

[1] Roopenian DC Akilesh S. FcRn: the neonatal Fc receptor comes of age. Nat Rev Immunol (2007) 7:715–25. 10.1038/nri2155 17703228

[2] Bourges D Meurens F Berri M Chevaleyre C Zanello G Levast B New insights into the dual recruitment of IgA+B cells in the developing mammary gland. Mol Immunol (2008) 45:3354–62. 10.1016/j.molimm.2008.04.017 18533264

[3] Norderhaug IN Johansen FE Schjerven H Brandtzaeg P. Regulation of the formation and external transport of secretory immunoglobulins. Crit Rev Immunol (1999) 19:481–508. 10647747

[4] Norderhaug IN Johansen FE Krajci P Brandtzaeg P. Domain deletions in the human polymeric Ig receptor disclose differences between its dimeric IgA and pentameric IgM interaction. Eur J Immunol (1999) 29:3401–9. 10.1002/(SICI)1521-4141(199910)29:10<3401::AID-IMMU3401>3.0.CO;2-G 10540352

[5] Hine B Hunt P Beasley A Windon R Glover S Colditz I. Selective transport of IgE into ovine mammary secretions. Res Vet Sci (2010) 89:184–90. 10.1016/j.rvsc.2010.02.010 20226487

[6] Qiao S-W Kobayashi K Johansen F-E Sollid LM Andersen JT Milford E Dependence of antibody-mediated presentation of antigen on FcRn. Proc Natl Acad Sci U S A (2008) 105:9337–42. 10.1073/pnas.0801717105 18599440 PMC2453734

[7] Baker K Rath T Pyzik M Blumberg RS. The role of FcRn in antigen presentation. Front Immunol (2014) 5:408. 10.3389/fimmu.2014.00408 25221553 PMC4145246

[8] Cui D Zhang L Li J Zhao Y Hu X Dai Y Bovine FcRn-mediated human immunoglobulin G transfer across the milk-blood barrier in transgenic mice. PLoS One (2014) 9:e115972. 10.1371/journal.pone.0115972 25546424 PMC4278800

[9] Schneider Z Jani PK Szikora B Vegh A Kovesdi D Ilias A Overexpression of bovine FcRn in mice enhances T-dependent immune responses by amplifying T helper cell frequency and Germinal Center Enlargement in the Spleen. Front Immunol (2015) 6:357. 10.3389/fimmu.2015.00357 26257730 PMC4507463

[10] Weflen AW Baier N Tang QJ Van den Hof M Blumberg RS Lencer WI Multivalent immune complexes divert FcRn to lysosomes by exclusion from recycling sorting tubules. Mol Biol Cell (2013) 24:2398–405. 10.1091/mbc.E13-04-0174 23741050 PMC3727932

[11] Kaetzel CS. The polymeric immunoglobulin receptor: bridging innate and adaptive immune responses at mucosal surfaces. Immunol Rev (2005) 206:83–99. 10.1111/j.0105-2896.2005.00278.x 16048543

[12] Asano M Komiyama K. Polymeric immunoglobulin receptor. J Oral Sci (2011) 53:147–56. 10.2334/josnusd.53.147 21712618

[13] Underdown BJ Schiff JM Immunoglobulin A: strategic defense initiative at the mucosal surface. Annu Rev Immunol (1986) 4:389–417. 10.1146/annurev.iy.04.040186.002133 3518747

[14] Vegh A Cervenak J Jankovics I Kacskovics I. FcRn overexpression in mice results in potent humoral response against weakly immunogenic antigen. MAbs (2011) 3:173–80. 10.4161/mabs.3.2.14462 21239891 PMC3092618

[15] Schneider Z Cervenak J Baranyi M Papp K Prechl J Laszlo G Transgenic expression of bovine neonatal Fc receptor in mice boosts immune response and improves hybridoma production efficiency without any sign of autoimmunity. Immunol Lett (2011) 137:62–9. 10.1016/j.imlet.2011.02.018 21338624

[16] Cervenak J Bender B Schneider Z Magna M Carstea BV Liliom K Neonatal FcR overexpression boosts humoral immune response in transgenic mice. J Immunol (2011) 186:959–68. 10.4049/jimmunol.1000353 21148035

[17] Wilson E Butcher EC. CCL28 controls immunoglobulin (Ig)A plasma cell accumulation in the lactating mammary gland and IgA antibody transfer to the neonate. J Exp Med (2004) 200:805–9. 10.1084/jem.20041069 15381732 PMC2211970

[18] Yoshida M Kobayashi K Kuo TT Bry L Glickman JN Claypool SM Neonatal Fc receptor for IgG regulates mucosal immune responses to luminal bacteria. J Clin Invest (2006) 116:2142–51. 10.1172/JCI27821 16841095 PMC1501111

[19] Osaka I Matsui Y Terada F. Effect of the mass of immunoglobulin (Ig) G intake and age at first colostrum feeding on serum IgG concentration in Holstein calves. J Dairy Sci (2014) 97(10):6608–12. 10.3168/jds.2013-7571 25064644

[20] Hurley WL Theil PK. Perspectives on immunoglobulins in colostrum and milk. Nutrients (2011) 3:442–74. 10.3390/nu3040442 22254105 PMC3257684

[21] Israel EJ Patel VK Taylor SF Marshak-Rothstein A Simister NE. Requirement for a beta 2-microglobulin-associated Fc receptor for acquisition of maternal IgG by fetal and neonatal mice. J Immunol (1995) 154:6246–51. 7759862

[22] Catunda Lemos AP Cervenak J Bender B Hoffmann OI Baranyi M Kerekes A Characterization of the rabbit neonatal Fc receptor (FcRn) and analyzing the immunophenotype of the transgenic rabbits that overexpresses FcRn. PLoS One (2012) 7:e28869. 10.1371/journal.pone.0028869 22247762 PMC3256154

[23] Lu W Zhao Z Zhao Y Yu S Zhao Y Fan B Over-expression of the bovine FcRn in the mammary gland results in increased IgG levels in both milk and serum of transgenic mice. Immunology (2007) 122:401–8. 10.1111/j.1365-2567.2007.02654.x 17608809 PMC2266012

[24] Adamski FM King AT Demmer J. Expression of the Fc receptor in the mammary gland during lactation in the marsupial *Trichosurus vulpecula* (brushtail possum). Mol Immunol (2000) 37:435–44. 10.1016/S0161-5890(00)00065-1 11090878

[25] Lemos AC Cervenak J Bender B Hoffmann OI Baranyi M Kerekes A Characterization of the rabbit neonatal Fc receptor (FcRn) and analyzing the immunophenotype of the transgenic rabbits that overexpresses FcRn. PLoS One (2012) 7:e28869. 10.1371/journal.pone.0028869 22247762 PMC3256154

[26] Verweij JJ Koets AP Eisenberg SW. Effect of continuous milking on immunoglobulin concentrations in bovine colostrum. Vet Immunol Immunopathol (2014) 160:225–9. 10.1016/j.vetimm.2014.05.008 24906350

[27] Suthar VS Canelas-Raposo J Deniz A Heuwieser W. Prevalence of subclinical ketosis and relationships with postpartum diseases in European dairy cows. J Dairy Sci (2013) 96:2925–38. 10.3168/jds.2012-6035 23497997

[28] Beam AL Lombard JE Kopral CA Garber LP Winter AL Hicks JA Prevalence of failure of passive transfer of immunity in newborn heifer calves and associated management practices on US dairy operations. J Dairy Sci (2009) 92:3973–80. 10.3168/jds.2009-2225 19620681

[29] Boumahrou N Chevaleyre C Berri M Martin P Bellier S Salmon H. An increase in milk IgA correlates with both pIgR expression and IgA plasma cell accumulation in the lactating mammary gland of PRM/Alf mice. J Reprod Immunol (2012) 96:25–33. 10.1016/j.jri.2012.08.001 23021255

[30] Macpherson AJ Geuking MB Slack E Hapfelmeier S Mccoy KD. The habitat, double life, citizenship, and forgetfulness of IgA. Immunol Rev (2012) 245:132–46. 10.1111/j.1600-065X.2011.01072.x 22168417

[31] Janoff EN Gustafson C Frank DN The world within: living with our microbial guests and guides. Transl Res (2012) 160:239–45. 10.1016/j.trsl.2012.05.005 22732305 PMC6440546

[32] Qiao S-W Sollid LM Blumberg RS. Antigen presentation in celiac disease. Curr Opin Immunol (2009) 21:111–7. 10.1016/j.coi.2009.03.004 19342211 PMC3901576

[33] Baker K Qiao S-W Kuo TT Aveson VG Platzer B Andersen J-T Neonatal Fc receptor for IgG (FcRn) regulates cross-presentation of IgG immune complexes by CD8− CD11b+ dendritic cells. Proc Natl Acad Sci U S A (2011) 108:9927–32. 10.1073/pnas.1019037108 21628593 PMC3116387

[34] Ye L Zeng R Bai Y Roopenian DC Zhu X. Efficient mucosal vaccination mediated by the neonatal Fc receptor. Nat Biotechnol (2011) 29:158–63. 10.1038/nbt.1742 21240266 PMC3197702

[35] Kacskovics I Cervenak J Erdei A Goldsby RA Butler JE. Recent advances using FcRn overexpression in transgenic animals to overcome impediments of standard antibody technologies to improve the generation of specific antibodies. MAbs (2011) 3:431–9. 10.4161/mabs.3.5.17023 22048692 PMC3225847

[36] Cervenak J Kurrle R Kacskovics I. Accelerating antibody discovery using transgenic animals overexpressing the neonatal Fc receptor as a result of augmented humoral immunity. Immunol Rev (2015) 268:269–87. 10.1111/imr.12364 26497527

[37] Baker K Rath T Flak MB Arthur JC Chen Z Glickman JN Neonatal Fc receptor expression in dendritic cells mediates protective immunity against colorectal cancer. Immunity (2013) 39:1095–107. 10.1016/j.immuni.2013.11.003 24290911 PMC3902970

[38] Uren TK Johansen FE Wijburg OL Koentgen F Brandtzaeg P Strugnell RA. Role of the polymeric Ig receptor in mucosal B cell homeostasis. J Immunol (2003) 170:2531–9. 10.4049/jimmunol.170.5.2531 12594279

[39] Rath T Kuo TT Baker K Qiao S-W Kobayashi K Yoshida M The immunologic functions of the neonatal Fc receptor for IgG. J Clin Immunol (2013) 33:9–17. 10.1007/s10875-012-9768-y 22948741 PMC3548031

[40] Yoshida M Claypool SM Wagner JS Mizoguchi E Mizoguchi A Roopenian DC Human neonatal Fc receptor mediates transport of IgG into luminal secretions for delivery of antigens to mucosal dendritic cells. Immunity (2004) 20:769–83. 10.1016/j.immuni.2004.05.007 15189741

[41] Suleiman YB Yoshida M Nishiumi S Tanaka H Mimura T Nobutani K Neonatal Fc receptor for IgG (FcRn) expressed in the gastric epithelium regulates bacterial infection in mice. Mucosal Immunol (2011) 5:87–98. 10.1038/mi.2011.53 22089027 PMC3964614

[42] Végh A Farkas A Kovesdi D Papp K Cervenak J Schneider Z FcRn overexpression in transgenic mice results in augmented APC activity and robust immune response with increased diversity of induced antibodies. PLoS One (2012) 7:e36286. 10.1371/journal.pone.0036286 22558422 PMC3340356

[43] Schneider Z Cervenak J Baranyi M Papp K Prechl J László G Transgenic expression of bovine neonatal Fc receptor in mice boosts immune response and improves hybridoma production efficiency without any sign of autoimmunity. Immunol Lett (2011) 137:62–9. 10.1016/j.imlet.2011.02.018 21338624

[44] Sait L Galic M Strugnell RA Janssen PH. Secretory antibodies do not affect the composition of the bacterial microbiota in the terminal ileum of 10-week-old mice. Appl Environ Microbiol (2003) 69:2100–9. 10.1128/AEM.69.4.2100-2109.2003 12676689 PMC154825

[45] Wijburg OL Uren TK Simpfendorfer K Johansen FE Brandtzaeg P Strugnell RA. Innate secretory antibodies protect against natural *Salmonella typhimurium* infection. J Exp Med (2006) 203:21–6. 10.1084/jem.20052093 16390940 PMC2118088

[46] Berry S Coppieters W Davis S Burrett A Thomas N Palmer D A triad of highly divergent polymeric immunoglobulin receptor (PIGR) haplotypes with major effect on IgA concentration in bovine milk. PLoS One (2013) 8:e57219. 10.1371/journal.pone.0057219 23536764 PMC3594236

[47] Sachs UJ Socher I Braeunlich CG Kroll H Bein G Santoso S A variable number of tandem repeats polymorphism influences the transcriptional activity of the neonatal Fc receptor α – chain promoter. Immunology (2006) 119:83–9. 10.1111/j.1365-2567.2006.02408.x 16805790 PMC1782336

[48] Zhang R Zhao Z Zhao Y Kacskovics I Eijk MVD Groot ND Association of FcRn heavy chain encoding gene (FCGRT) polymorphisms with IgG content in bovine colostrum. Anim Biotechnol (2009) 20:242–6. 10.1080/10495390903196448 19937499

[49] Obara W Iida A Suzuki Y Tanaka T Akiyama F Maeda S Association of single-nucleotide polymorphisms in the polymeric immunoglobulin receptor gene with immunoglobulin A nephropathy (IgAN) in Japanese patients. J Hum Genet (2003) 48:293–9. 10.1007/s10038-003-0027-1 12740691

[50] Kim J Bronson C Wani MA Oberyszyn TM Mohanty S Chaudhury C β2-Microglobulin deficient mice catabolize IgG more rapidly than FcRn-α-chain deficient mice. Exp Biol Med (2008) 233:603–9. 10.3181/0710-RM-270 18375831

[51] Bender B Bodrogi L Mayer B Schneider Z Zhao Y Hammarstrom L Position independent and copy-number-related expression of the bovine neonatal Fc receptor alpha-chain in transgenic mice carrying a 102 kb BAC genomic fragment. Transgenic Res (2007) 16:613–27. 10.1007/s11248-007-9108-9 17594529

[52] Robert-Guroff M IgG surfaces as an important component in mucosal protection. Nat Med (2000) 6:129–30. 10.1038/72206 10655090

[53] De Groot N Van Kuik-Romeijn P Lee SH De Boer HA Increased immunoglobulin A levels in milk by over – expressing the murine polymeric immunoglobulin receptor gene in the mammary gland epithelial cells of transgenic mice. Immunology (2000) 101:218–24. 10.1046/j.1365-2567.2000.00094.x 11012775 PMC2327069

[54] Johansen F-E Pekna M Norderhaug IN Haneberg B Hietala MA Krajci P Absence of epithelial immunoglobulin a transport, with increased mucosal leakiness, in polymeric immunoglobulin receptor/secretory component–deficient mice. J Exp Med (1999) 190:915–22. 10.1084/jem.190.7.915 10510081 PMC2195652

[55] Hendrickson BA Rindisbacher L Corthesy B Kendall D Waltz DA Neutra MR Lack of association of secretory component with IgA in J chain-deficient mice. J Immunol (1996) 157:750–4. 8752925

[56] van IJzendoorn SC Tuvim MJ Weimbs T Dickey BF Mostov KE. Direct interaction between Rab3b and the polymeric immunoglobulin receptor controls ligand-stimulated transcytosis in epithelial cells. Dev Cell (2002) 2:219–28. 10.1016/S1534-5807(02)00115-6 11832247

[57] Macpherson AJ McCoy KD Johansen FE Brandtzaeg P. The immune geography of IgA induction and function. Mucosal Immunol (2008) 1:11–22. 10.1038/mi.2007.6 19079156

[58] Johansen FE Kaetzel CS. Regulation of the polymeric immunoglobulin receptor and IgA transport: new advances in environmental factors that stimulate pIgR expression and its role in mucosal immunity. Mucosal Immunol (2011) 4:598–602. 10.1038/mi.2011.37 21956244 PMC3196803

[59] Pal K Kaetzel CS Brundage K Cunningham CA Cuff CF. Regulation of polymeric immunoglobulin receptor expression by reovirus. J Gen Virol (2005) 86:2347–57. 10.1099/vir.0.80690-0 16033983

[60] Priestley D Bittar J Ibarbia L Risco C Galvao K. Effect of feeding maternal colostrum or plasma-derived or colostrum-derived colostrum replacer on passive transfer of immunity, health, and performance of preweaning heifer calves. J Dairy Sci (2013) 96:3247–56. 10.3168/jds.2012-6339 23497992

[61] Rogier EW Frantz AL Bruno MEC Leia W Cohen DA Stromberg AJ Secretory antibodies in breast milk promote long-term intestinal homeostasis by regulating the gut microbiota and host gene expression. Proc Natl Acad Sci U S A (2014) 111:3074–9. 10.1073/pnas.1315792111 24569806 PMC3939878

[62] Lara-Padilla E Campos-Rodriguez R Jarillo-Luna A Reyna-Garfias H Rivera-Aguilar V Miliar A Caloric restriction reduces IgA levels and modifies cytokine mRNA expression in mouse small intestine. J Nutr Biochem (2011) 22:560–6. 10.1016/j.jnutbio.2010.04.012 20951020

[63] Cervenak J Doleschall M Bender B Mayer B Schneider Z Doleschall Z NFκB induces overexpression of bovine FcRn: a novel mechanism that further contributes to the enhanced immune response in genetically modified animals carrying extra copies of FcRn. MAbs (2013) 5(6):860–71. 10.4161/mabs.26507 24492342 PMC3896600

[64] Cox S Ebersole L Carpenter G Proctor G. Effects of autonomic agonists and immunomodulatory cytokines on polymeric immunoglobulin receptor expression by cultured rat and human salivary and colonic cell lines. Arch Oral Biol (2007) 52:411–6. 10.1016/j.archoralbio.2006.10.006 17118334

[65] Brandtzaeg P Role of secretory antibodies in the defence against infections. Int J Med Microbiol (2003) 293:3–15. 10.1078/1438-4221-00241 12755363

[66] Pine R. Convergence of TNF alpha and IFN gamma signalling pathways through synergistic induction of IRF-1/ISGF-2 is mediated by a composite GAS/kappa B promoter element. Nucleic Acids Res (1997) 25:4346–54. 10.1093/nar/25.21.4346 9336467 PMC147058

[67] Stark A Vachkova E Wellnitz O Bruckmaier R Baumrucker C. Colostrogenesis: candidate genes for IgG1 transcytosis mechanisms in primary bovine mammary epithelial cells. J Anim Physiol Anim Nutr (2013) 97:1114–24. 10.1111/jpn.12021 23279563

